# Preclinical Evaluation of [^68^Ga]Ga-DFO-ZEGFR:2377: A Promising Affibody-Based Probe for Noninvasive PET Imaging of EGFR Expression in Tumors

**DOI:** 10.3390/cells7090141

**Published:** 2018-09-18

**Authors:** Maryam Oroujeni, Javad Garousi, Ken G. Andersson, John Löfblom, Bogdan Mitran, Anna Orlova, Vladimir Tolmachev

**Affiliations:** 1 Department of Immunology, Genetics and Pathology, Uppsala University, SE- 751 85 Uppsala, Sweden; maryam.oroujeni@igp.uu.se (M.O.); javad.garousi@igp.uu.se (J.G.); 2 Department of Protein Science, School of Engineering Sciences in Chemistry, Biotechnology and Health, KTH Royal Institute of Technology, SE- 106 91 Stockholm, Sweden; ken2@kth.se (K.G.A.); lofblom@kth.se (J.L.); 3 Department of Medicinal Chemistry, Uppsala University, SE- 751 23 Uppsala, Sweden; mitran.bogdan@ilk.uu.se (B.M.); anna.orlova@ilk.uu.se (A.O.); 4 Science for Life Laboratory, Uppsala University, SE- 750 03 Uppsala, Sweden

**Keywords:** EGFR, radionuclide imaging, PET, affibody molecules, Ga-68, Zr-89, DFO, nude mice, A431 xenografts

## Abstract

Radionuclide imaging of epidermal growth factor receptor (EGFR) expression in tumors may stratify patients for EGFR-targeting therapies and predict response or resistance to certain treatments. Affibody molecules, which are nonimmunoglobulin scaffold proteins, have a high potential as probes for molecular imaging. In this study, maleimido derivative of desferrioxamine B (DFO) chelator was site-specifically coupled to the C-terminal cysteine of the anti-EGFR affibody molecule ZEGFR:2377, and the DFO-ZEGFR:2377 conjugate was labeled with the generator-produced positron-emitting radionuclide ^68^Ga. Stability, specificity of binding to EGFR-expressing cells, and processing of [^68^Ga]Ga-DFO-ZEGFR:2377 by cancer cells after binding were evaluated in vitro. In vivo studies were performed in nude mice bearing human EGFR-expressing A431 epidermoid cancer xenografts. The biodistribution of [^68^Ga]Ga-DFO-ZEGFR:2377 was directly compared with the biodistribution of [^89^Zr]Zr-DFO-ZEGFR:2377. DFO-ZEGFR:2377 was efficiently (isolated yield of 73 ± 3%) and stably labeled with ^68^Ga. Binding of [^68^Ga]Ga-DFO-ZEGFR:2377 to EGFR-expressing cells in vitro was receptor-specific and proportional to the EGFR expression level. In vivo saturation experiment demonstrated EGFR-specific accumulation of [^68^Ga]Ga-DFO-ZEGFR:2377 in A431 xenografts. Compared to [^89^Zr]Zr-DFO-ZEGFR:2377, [^68^Ga]Ga-DFO-ZEGFR:2377 demonstrated significantly (*p* < 0.05) higher uptake in tumors and lower uptake in spleen and bones. This resulted in significantly higher tumor-to-organ ratios for [^68^Ga]Ga-DFO-ZEGFR:2377. In conclusion, [^68^Ga]Ga-DFO-ZEGFR:2377 is a promising probe for imaging of EGFR expression.

## 1. Introduction

Radionuclide molecular imaging may provide accurate assessment of expression of molecular targets in disseminated cancer and enable selection of patients for targeted treatment. The imaging agents should provide a high imaging contrast to ensure sufficient sensitivity of imaging. Off-target interactions may result in appreciable accumulation of probes in healthy tissues and reduce imaging contrast. Selection of an optimal combination of a targeting protein—a radionuclide with suitable physical and chemical properties and a chelator or a linker for coupling of the radionuclide—is critical to minimize off-target interactions and guarantee the best possible contrast [1]. This work is dedicated to finding an optimal affibody-based probe for visualization of one of the more challenging imaging targets—the epidermal growth factor receptor (EGFR; other designations: HER1or ErbB1).

Epidermal growth factor receptor is a transmembrane tyrosine kinase receptor belonging to the ErbB (HER) family of receptors. Activation of EGFR triggers intracellular signaling that can lead to cell proliferation, increased motility, and inhibition of apoptosis [2]. Overexpression of EGFR is associated with resistance to chemotherapy and radiotherapy and poor prognosis in many cancers, such as head and neck squamous cell carcinoma (HNSCC) [3], breast cancer [4], and non-small-cell lung cancer (NSCLC) [5]. Response to various EGFR-targeting strategies for treatments of HNSCC and NSCLC is associated with a high level of EGFR expression [6,7,8]. Thus, detection of EGFR expression level in malignant tumors can provide important prognostic and predictive information that can influence the stratification of cancer treatment for patients. 

The use of biopsy samples is a common method to determine the level of EGFR expression in human tumors. However, this methodology is associated with several problems, such as invasiveness, alteration of expression level during the disease progression, a discordant expression in primary tumors and metastases, and sampling errors [9,10,11]. Visualization of EGFR expression using molecular imaging techniques, such as single photon emission computed tomography (SPECT) and positron-emission tomography (PET), and EGFR-specific probes can overcome many problems of biopsy-based methods. Simultaneous evaluation of expression levels in both the primary tumor and the metastases is possible in this way. In addition, these imaging techniques can provide repetitive noninvasive determination of the level of EGFR expression in tumors. The use of these techniques would make cancer treatment more personalized. 

Several types of antibody-based imaging probes have been evaluated for imaging EGFR, such as the anti-EGFR monoclonal antibody panitumumab labeled with ^89^Zr [12,13] or ^86^Y [14], F(ab´)_2_ fragment of panitumumab radiolabeled with either ^111^In or ^86^Y [15], and the anti-EGFR monoclonal antibody cetuximab labeled with ^89^Zr [16] or ^64^Cu [17]. The major issue with the use of monoclonal antibodies for imaging is their slow clearance from blood and nonspecific compartments, which requires a long time (6–7 days) [18] to obtain an acceptable imaging contrast. As an alternative, the natural ligand—epidermal growth factor (EGF)—labeled with ^111^In [19], ^131^I [20], ^64^Cu [21], ^18^F [22] or ^68^Ga [23] has been evaluated. A common issue for all imaging probes is an endogenous expression of EGFR in a number of healthy tissues. Of particular concern is the rather high EGFR expression in hepatocytes (approximately 600,000 receptors per cell [24]), which may sequester a probe from circulation [25]. Fortunately, it has been shown that for several classes of imaging agents, an increase in the injected protein dose by adding nonlabeled imaging probe enables the saturation of EGFR in liver without saturation in tumors [22,25,26]. However, this approach is not applicable to EGF-based imaging probes because the strong physiologic action of the ligand caused severe adverse side effects (hypotension, diarrhea, nausea, and vomiting) during clinical dose escalation studies [20].

Affibody molecules are very promising targeting proteins for development of probes for in vivo radionuclide imaging [27,28]. Affibody molecules are nonimmunoglobulin affinity proteins that are based on the three-helix bundle domain B of protein A. Thirteen amino acids in helices 1 and 2 are randomized, which creates a large library that permits selection of high-affinity binders to different molecular structures, including cancer-associated targets [27]. Affibody molecules (molecular weight of 6–7 kDa) are much smaller than bulky monoclonal antibodies (150 kDa). Due to their small size, they demonstrate an efficient extravasation and a rapid tumor penetration. The unbound probe is rapidly cleared from blood via the kidneys, which makes it possible to obtain high contrast images after only a few hours after injection [28]. Several affibody-based EGFR-specific probes have previously been labeled with radionuclides, such as ^99m^Tc [29] and ^111^In [26] for SPECT, and ^18^F [30,31], ^55/57^Co [32], ^68^Ga [32], ^64^Cu [33,34], and ^89^Zr [35,36] for PET imaging applications.

PET is an imaging method that can provide superior sensitivity and quantitation accuracy compared to SPECT [37]. Gallium-68 (^68^Ga, T_1/2_ = 67.6 min, β^+^ abundance 90%, E β+max = 1880 keV) is a suitable positron-emitting radionuclide for clinical PET imaging. The advantages of this radionuclide include the good availability of gallium-68 because of generator production and the short half-life of 67.6 min, which results in low absorbed dose burden to patients. These advantages have resulted in great interest in gallium-68-labeled radiopharmaceuticals, especially the ones based on rapidly cleared peptides [38,39].

An assessment of anti-EGFR affibody molecules ZEGFR:2377 labeled with gallium-68 via the commonly used DOTA (1,4,7,10-Tetraazacyclododecane-1,4,7,10-tetraacetic acid); chelator showed a high liver uptake (6.2 ± 0.3% ID/g) and relatively low tumor uptake (2.7 ± 0.1% ID/g) 3 h after injection [32]. In addition, the clearance of [^68^Ga]Ga-DOTA-ZEGFR:2377 from blood was found to be slower compared to the clearance of ^68^Ga-labeled affibody molecules for imaging of other molecular targets. Such biodistribution pattern is typical for derivatives of ZEGFR:2377. This affibody molecule has been selected to have equal subnanomolar affinity to human and murine EGFR [26] in order to make murine models realistic and provide adequate data for clinical translation. As the internalization of affibody molecules after binding to EGFR is slow [26,32,35], the majority of the specifically bound tracer remains on the cell surface of both tumor cells and hepatocytes. The rapid renal clearance of the probe shifts the binding equilibrium to dissociation. Thus, the liver—acting as a depot for anti-EGFR-affibody molecules—releases molecules into the blood stream, causing slower blood clearance compared with other affibody molecules. Therefore, the focus in the search for an optimal positron-emitted label for anti-EGFR affibody molecules was shifted to more long-lived positron emitters, such as ^55^Co (T_1/2_ = 17.5 h) [32], ^64^Cu (T_1/2_ = 12.7 h) [33,34], and ^89^Zr (T_1/2_ = 78.4 h) [35,36], which would provide PET imaging the next day after injection. These studies resulted in interesting findings. It was found that a combination of DOTA chelators and radionuclide (^68^Ga or ^57^Co) has very substantial impact on hepatic uptake of ZEGFR:2377 [32]. This indicated that besides EGFR-dependent mechanism of uptake ZEGFR:2377 in liver, there is an EGFR-independent mechanism that can be influenced by the charge of a chelator–metal complex [32]. Comparison of siderophore-based chelators fusarinine C (FSC) and desferrioxamine B (DFO) for labeling of ZEGFR:2377 with ^89^Zr also revealed strong influence of a radionuclide–chelator combination on biodistribution properties [36]. In addition, it was shown that labeling of DFO-ZEGFR:2377 with ^89^Zr at elevated temperature (85 °C) enabled appreciable improvement in the imaging properties of the conjugate, presumably due to higher stability of the chelate [36].

**Figure 1:**
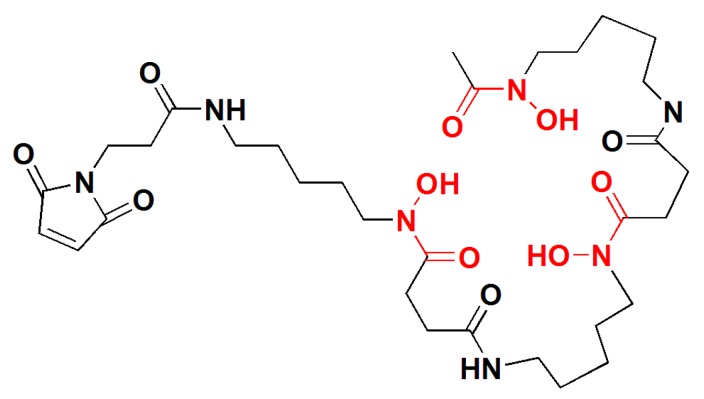
Structure of maleimide-derivatized desferrioxamine B (DFO).

For site-specific labeling of ZEGFR:2377 with ^89^Zr, a maleimido derivative of desferrioxamine B (*N*-[5-[[4-[5-[acetyl(hydroxy)amino]pentylamino]-4-oxobutanoyl]-hydroxyamino]pentyl]-*N*′-(5-aminopentyl)-*N*′-hydroxybutanediamide, DFO) was conjugated to unique C-terminal cysteine of this affibody molecule (Figure 1). Several studies have shown that DFO is a suitable chelator for radiolabeling of biomolecules with gallium-68 [40,41]. In fact, DFO is one of the first chelators used for labeling with [^67/68^Ga]Ga^3+^ with high radiochemical yields [42]. DFO is an acyclic hexadentate chelator based on a siderophore from the bacteria *Streptomyces pilosus*. It contains three hydroxamate groups, which provide six oxygen donors for the metal binding [43]. This is a good match for Ga^3+^, which is typically hexacoordinated. In addition, the use of Ga^3+^ instead of Zr^4+^ decreases the positive charge on C-terminus of ZEGFR:2377, which is favorable for biodistribution [32,44]. 

The goal of this study was to evaluate targeting properties of [^68^Ga]Ga-DFO-ZEGFR:2377 labeled at elevated temperature and compare them directly with the properties of [^89^Zr]Zr-DFO-ZEGFR:2377. For this purpose, labeling of DFO-ZEGFR:2377 affibody molecules with gallium-68 was optimized stability and in vitro and in vivo targeting properties of [^68^Ga]Ga-DFO-ZEGFR:2377 was investigated.

## 2. Materials and Methods

### 2.1. Materials and Equipment

Gallium-68 was obtained by fractionated elution of the ^68^Ge/^68^Ga generator (Eckert and Ziegler AG, Berlin, Gemany) with 0.1 M HCl. The eluate with the highest radioactivity concentration was used for labeling. Zirconium-89 (solution in 1 M oxalic acid) was purchased from PerkinElmer (Waltham, MA, USA). The radioactivity was measured using a gamma spectrometer with a NaI(Tl) detector (1480 WIZARD, Wallac Oy, Turku, Finland). The labeling yield and radiochemical purity of labeled conjugates were measured using the instant thin-layer chromatography (ITLC) (ITLC-SG, Agilent Technologies, Santa Clara, CA, USA). The strips were developed with 0.2 M citric acid, pH 2.0 and 5 for gallium-68 and zirconium-89 conjugates, respectively. Distribution of the radioactivity among the strips was measured on Cyclone Phosphor Storage Screen using OptiQuant software for data processing (both Packard Instrument Company, Meriden, CT, USA) as well as Fujifilm Bioimaging Analyzers (BAS) 1800II using MultiGauge V3.0 analysis software. Radiolabeled bioconjugates were purified for further studies using phosphate-buffered saline (PBS) pre-equilibrated with 2% bovine serum albumin using NAP-5 size exclusion column (GE Healthcare, Stockholm, Sweden). 

**Figure 2:**
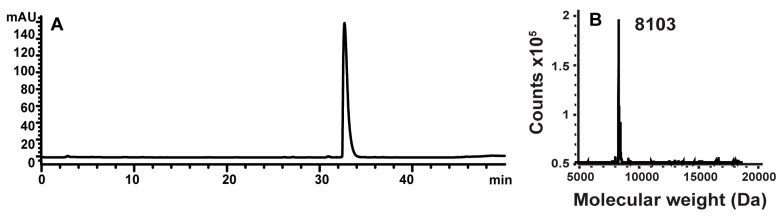
(**A**) HPLC chromatogram (**B**) mass spectrum of DFO-ZEGFR:2377 conjugate. Expected mass is 8102.8 Da.

The anti-EGFR affibody molecules containing C-terminal cysteine was produced as described earlier [26]. Maleimido-DFO (Figure 1), was site-specifically conjugated to C-terminal cysteine of ZEGFR:2377 to provide DFO-ZEGFR:2377 according to Garousi and co-workers [35]. The identity of conjugates was confirmed by mass spectrometry as described earlier [35]. The purity of the conjugate was over 95%, as determined using reversed phase HPLC according to a method described earlier [35] (Figure 2). The conjugate was aliquoted and freeze dried. 

Statistical treatment and linear regression analysis were performed using GraphPad Prism software version 5.00 for Windows, GraphPad Software, San Diego, CA, USA. A two-tailed unpaired *t*-test was used for comparison of the two sets of data. The difference was considered as significant when *p* value was less than 0.05. 

### 2.2. Radiolabeling and In Vitro Stability

For labeling with ^68^Ga, DFO-ZEGFR:2377 was reconstituted in 10% ethanol in water to obtain a concentration of 2 mg/mL. Then, the DFO-ZEGFR:2377 solution (30 μg, 15 μL) was mixed with 80 μL of 1.25 M of NaOAc, pH 4. A generator eluate (120 μL, 90–100 MBq) was added; the mixture was thoroughly vortexed and incubated for 10 min either at room temperature or at 85 °C.

DFO-ZEGFR:2377 was labeled with zirconium-89 according to the following protocol [36]: Zirconium-89 solution (8.7 μL, 10−12 MBq) was neutralized by adding 8.3 μL of Na_2_CO_3_ (1 M) and incubated for 3 min at RT. Then, 66.7 μL of HEPES (2-(4-(2-Hydroxyethyl)piperazin-1-yl)ethane-sulfonic acid) buffer (0.5 M, pH 6.98) was added to the radionuclide solution. After addition of 20 μL of bioconjugate solution (1 μg/μL in 10% *v*/*v* EtOH in water), the resulting mixture was incubated 30 min at 85 °C.

To estimate the total mass of the conjugate after purification and to calculate apparent specific radioactivity, the activity of the conjugate before purification and the activity of the purified conjugate was measured using the dose calibrator VDC-405 (Veenstra Instruments BV, Joure, The Netherlands). Based on this data and data concerning radiochemical purity of the conjugate before and after purification, the percentage of the radiolabeled conjugate lost during purification was calculated. The mass of unlabeled conjugate was calculated with the assumption that the losses were equal for both labeled and unlabeled conjugate.

In vitro stability of [^68^Ga]Ga-DFO-ZEGFR:2377 was evaluated in PBS and in the presence of 1000-fold molar excess of EDTA (ethylenediaminetetraacetic acid). After purification, samples of freshly labeled conjugate (1.4 μg, 50 μL) were mixed with EDTA (64 μg, 2.5 mg/mL in PBS) to obtain a 1000-fold molar excess of EDTA. Control samples were mixed with equal volume of PBS. The experiment was performed in triplicate.

### 2.3. In Vitro Studies

For cell studies, two EGFR-expressing cell lines with different level of EGFR expression—epidermoid carcinoma A431 (1.2 × 10^6^ receptors/cell) [45] and prostate carcinoma PC3 (10^5^ receptors/cell) [46] (ATCC)—were used. The cell lines were cultured in McCoy’s medium, supplemented with 10% fetal bovine serum (Sigma-Aldrich, St. Louis, MO, USA ), 1% l-glutamine, and PEST (penicillin 100 μg/mL and 100 μg/mL streptomycin), from Bookroom AG (Berlin, Germany). The cells were cultured at 37 °C in a humidified incubator with 5% CO_2_.

For a binding specificity test, a set of three dishes containing approximately 10^6^ cells/dish was used for each data point. To saturate EGF receptors, a 100-fold molar excess of anti-EGFR antibody cetuximab and anti-EGFR affibody His_6_-ZEGFR:2377 were used. For control, nonbinding anti-CAIX affibody HE_3_-ZCAIX, and anti-VEGF-A bevacizumab were used. Cells were incubated with blocking agents at room temperature for 15 min. Then, [^68^Ga]Ga-DFO-ZEGFR:2377 (5 nM) was added to all dishes followed by incubation for 1 h at 37 °C. After incubation, the medium was aspired. Then, the cells were washed with cold serum-free medium, treated with 2 mL trypsin–EDTA solution per dish at 37 °C and collected. Radioactivity of cells was measured. The experiments were performed in triplicate.

Cellular processing of bound [^68^Ga]Ga-DFO-ZEGFR:2377 was evaluated using A431 and PC3 cells. The cells (10^6^ cells/dish) were incubated with 5 nM of [^68^Ga]Ga-DFO-ZEGFR:2377 at 37 °C. After 1, 2, and 3 h incubation, the internalized fraction was determined by an acid wash method [47]. The membrane-bound affibody molecules were removed from cells by treatment with 4 M urea solution in a 0.1 M glycine buffer, pH 2.5, for 5 min on ice. The cell debris containing the internalized conjugates was detached by treatment with 1 M NaOH. Radioactivity of samples was measured, and percentage of membrane-bound, internalized and total radioactivity was calculated. The experiments were performed in triplicate.

The antigen-binding capacity (ABC) was analyzed according to the method described by Engfeldt et al. [48]. A431 cells were scraped from six culture flasks, and six cell pellets containing 10^7^ cells were formed in Eppendorf tubes by gentle centrifugation. The supernatant was discarded. One milliliter of 10 nM solution of [^68^Ga]Ga-DFO-ZEGFR:2377 in cell culture medium was added to each pellet to provide approximately 100-fold calculated molar excess of receptor over conjugate. The cells were gently re-suspended and incubated for 2 h at 4 °C under slight shaking. After incubation, cells were pelleted, and 0.5 mL of the supernatant was transferred to Eppendorf tubes. The radioactivity of the samples containing pelleted cells and supernatant were measured, and the ABC was calculated.

### 2.4. In Vivo Studies

Animal studies were planned and performed in agreement with EU Directive 2010/63/EU for animal experiments and Swedish national legislation concerning protection of laboratory animals. Experiments were approved by the Ethics Committee for Animal Research in Uppsala (C4/16).

Biodistribution studies were performed in female BALB/C nu/nu mice purchased from Scanbur A/S (Karlslunde, Denmark). 

EGFR-expressing xenografts were established by subcutaneous injection of 10^7^ A431 cells in the left hind legs of mice. The tumors were grown for 10–14 days. The animals were randomized into groups of at least four mice for each data point. At the time of the experiment, the average animal weight was 18 ± 1 g. Average tumor weight was 0.6 ± 0.4 g. The in vivo specificity of conjugates was investigated using saturation of EGF receptors in a group of mice by subcutaneous injection of 10 mg of cetuximab 24 h before the injection of radiolabeled affibody molecules. To measure biodistribution, mice were intravenously (tail vein) injected with 800 kBq (38 μg) of [^68^Ga]Ga-DFO-ZEGFR:2377 in 100 μL PBS. For comparison, a group of mice was intravenously injected with 40 kBq (38 μg) of [^89^Zr]Zr-DFO-ZEGFR:2377 in 100 μL PBS. The injected activity was selected based on results of previous studies to provide counting statistics error of less than 3% for samples with the lowest uptake and the dead time of less than 20% for the standards of injected activity. The injected protein dose was determined in earlier studies as optimal for a partial saturation of EGFR in liver without saturation of receptors in tumor [26]. To obtain the desirable protein dose, an unlabeled DFO-ZEGFR:2377 was added to conjugates during formulation.

The biodistribution was measured 3 h postinjection (pi), as this is a clinically meaningful time point for ^68^Ga-labeled tracers. The mice were euthanized by an intraperitoneal injection of anesthetic solution (20 μL of solution per gram of body weight: Ketalar, 10 mg/mL; Rompun, 1 mg/mL) followed by heart puncture. Blood, salivary glands, lung, liver, spleen, colon, kidneys, tumor, muscle, and bone were collected and weighed. Then, the organ radioactivity was measured and uptake values of organs were calculated as percentage injected dose per gram tissue (% ID/g). 

Whole body positron emission tomography (PET)/computed tomography (CT) scans of the mice injected with [^68^Ga]Ga-DFO-ZEGFR:2377 (35 μg, 5 MBq) or [^89^Zr]Zr-DFO-ZEGFR:2377 (35 μg, 5 MBq) were performed under general anesthesia in nanoScan PET/MRI (Mediso Medical Imaging Systems Ltd., Budapest, Hungary) 3 h after injection. Anesthesia was induced and maintained by the administration of a mixture of sevoflurane (2.5–3.5%), oxygen, and medical air. CT acquisition was performed using nanoScan SPECT/CT (Mediso Medical Imaging Systems Ltd., Hungary) immediately after PET acquisition using the same bed position. The PET scans were performed for 30 min, followed by CT examination at the following parameters: CT-energy peak of 50 keV, 670 μA, 480 projections, 2.29 min acquisition time. The PET data were reconstructed into a static image using Tera-Tomo™ 3D reconstruction engine. CT raw files were reconstructed in real time using Filter Back Projection in Nucline 2.03 Software (Mediso Medical Imaging Systems, Hungary). PET and CT files were fused and analyzed using Nucline 2.03 Software (Mediso Medical Imaging Systems, Hungary) and were presented as maximum intensity projections (MIP) in RGB color scale.

## 3. Results

### 3.1. Radiolabeling and In Vitro Stability

**Figure 3:**
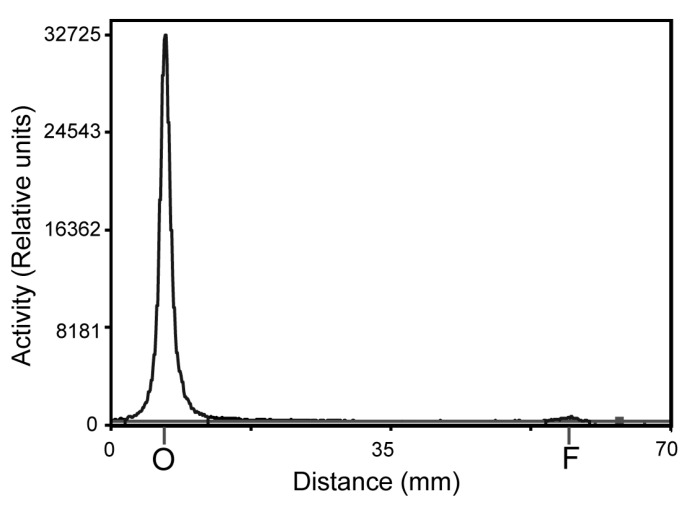
Distribution of activity of purified [^68^Ga]Ga-DFO-ZEGFR:2377 labeled at 85 °C along ITLC strip. Retardation factor of affibody molecule is 0.0 and that of free ^68^Ga is 1.0.

The gallium-68 labeling of ZEGFR:2377 using DFO as a chelator was performed at room temperature room (RT) and 85 °C. Table 1 shows the results of labeling experiments. The radiochemical yield for the labeled conjugate at RT was higher than the labeled conjugate at 85 °C. However, the isolated yield for DFO-ZEGFR:2377 labeled at RT did not differ significantly from the yield after labeling at 85 °C. The radiochemical purity of the radiolabeled affibody molecules after purification using NAP-5 columns was above 98% for both conjugates labeled at RT and 85 °C (Figure 3).

Zirconium-89 labeling of DFO-ZEGFR:2377 was efficiently performed according to the protocol of Summer and co-workers [36].

After the purification, in vitro stability test of DFO-ZEGFR:2377 labeled with gallium-68 at RT and 85 °C was performed in PBS and in the presence of 1000-fold molar excess of EDTA. The results of the in vitro stability test (Table 2) demonstrated that DFO-ZEGFR:2377 labeled with gallium-68 at both RT and elevated temperature was stable in PBS and under EDTA challenge (>97%). 

### 3.2. In Vitro Studies

**Figure 4:**
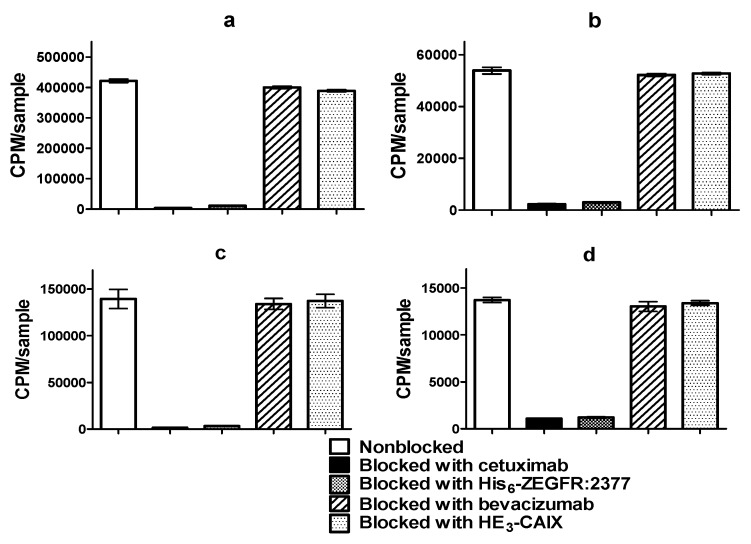
In vitro specificity of [^68^Ga]Ga-DFO-ZEGFR:2377 conjugate labeled at RT bound to (**a**) A431 and (**b**) PC3; labeled at 85 °C bound to (**c**) A431 and (**d**) PC3. Cells were incubated with 5 nM [^68^Ga]Ga-DFO-ZEGFR:2377. 500 nM of blocking agents was used to check the epidermal growth factor (EGF) receptor-specific of the conjugate. The data are presented as the average (n = 3) and SD.

The specificity of [^68^Ga]Ga-DFO-ZEGFR:2377 binding to EGFR expressing cells was tested by presaturation of receptors. Saturation of the receptors with a large excess of anti-EGFR antibody cetuximab and anti-EGFR affibody molecule ZEGFR:2377 (His_6_-ZEGFR:2377) significantly (*p* < 0.00005) decreased the binding of the radiolabeled affibody molecules to EGFR-expressing cell lines A431 as well as PC3 cells. Therefore, specificity test of DFO-ZEGFR:2377 labeled at RT and 85 °C demonstrated that the binding of the tracer to both EGFR-expressing cell lines was EGF receptor mediated. In addition, cell-associated activity was proportional to EGFR expression (Figure 4).

The treatment of cells with anti-VEGF-A antibody bevacizumab and anti-CAIX affibody—HE_3_-ZCAIX—did not show any influence on binding of the radiolabeled affibody molecules. This suggests that the binding of [^68^Ga]Ga-DFO-ZEGFR:2377 to cells was not unspecifically influenced by an affibody scaffold or nonbinding domains of antibodies. These experiments demonstrated that the binding of all [^68^Ga]Ga-DFO-ZEGFR:2377-labeled conjugates was EGFR-specified.

**Figure 5:**
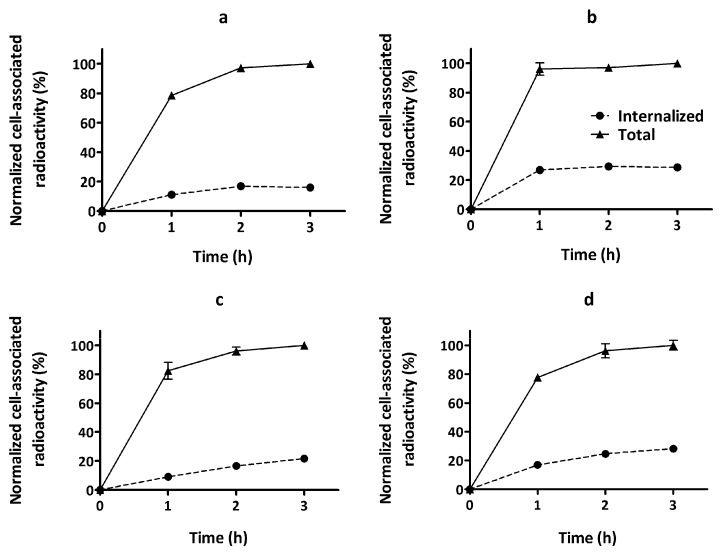
Cellular processing of DFO-ZEGFR:2377 labeled with gallium-68 (**a**) at RT by A431, (**b**) at RT by PC3, (**c**) at 85 °C by A431, and (**d**) at 85 °C by PC3. Cells were incubated with 5 nM [^68^Ga]Ga-DFO-ZEGFR:2377 conjugate. Cell-associated activity was normalized to the maximum uptake. The data are presented as average (n = 3) and SD. Error bars might not been seen because they are smaller than the symbols.

The processing of bound [^68^Ga]Ga-DFO-ZEGFR:2377 by A431 and PC3 cell lines was studied by the acid wash method. The data are presented in Figure 5. The labeled conjugate showed a rapid binding but was relatively slowly internalized, which is typical for affibody molecules. The internalized pattern was similar in both cell lines and was practically independent of labeling temperature. 

The antigen-binding capacity measurements showed that ABC of DFO-ZEGFR:2377 labeled with gallium-68 at RT (78.2 ± 0.4%) was slightly but significantly (*p* < 0.05) higher than the ABC of a conjugate labeled at elevated temperature (74.0 ± 0.5%).

### 3.3. In Vivo Studies

**Figure 6:**
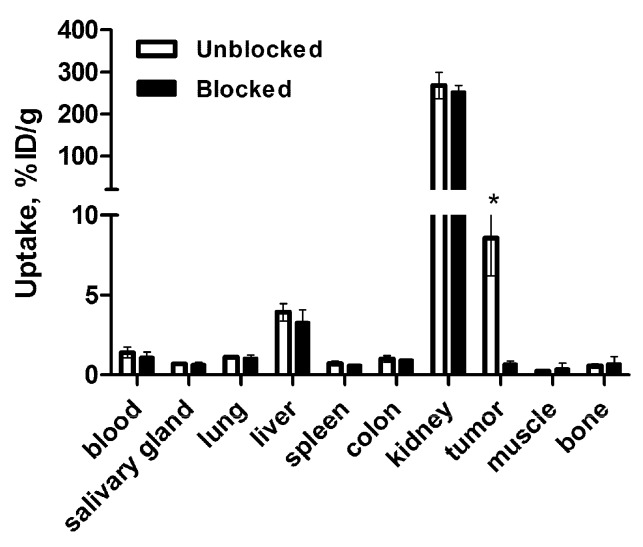
In vivo specificity of [^68^Ga]Ga-DFO-ZEGFR:2377 accumulation in A431 xenografts in mice 3 h after injection. In the blocked group, receptors were saturated by preinjection of large excess of anti-EGFR antibody cetuximab 24 h before the experiment. The data were presented as the average (n = 4 for blocked group and n = 7 for unblocked group) and SD. Asterisk (*) marks significant (*p* < 0.0005) difference.

The in vivo specificity of [^68^Ga]Ga-DFO-ZEGFR:2377 binding to EGFR in tumor xenografts was evaluated by presaturation of receptors using the monoclonal antibody cetuximab in tumor (Figure 6). Tumor uptake in the blocked group was significantly (*p* < 0.0005) reduced compared to unblocked group, indicating highly specific accumulation of [^68^Ga]Ga-DFO-ZEGFR:2377 in tumors.

**Figure 7:**
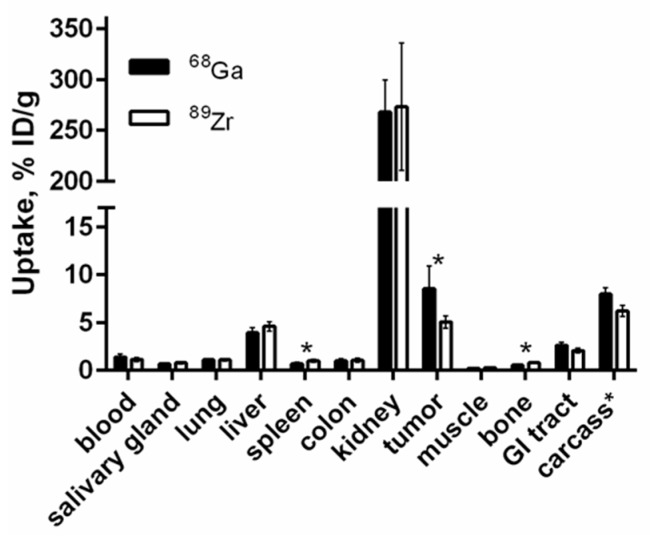
Biodistribution of [^68^Ga]Ga-DFO-ZEGFR:2377 and [^89^Zr]Zr-DFO-ZEGFR:2377 conjugates in BALB/C nu/nu mice bearing EGFR-expressing A431 xenografts 3 h after injection. The data were presented as the average (n = 7) and SD. Error bars might not be seen because they are smaller than the symbols. Asterisk (*) marks significant (*p* < 0.05) difference.

**Figure 8:**
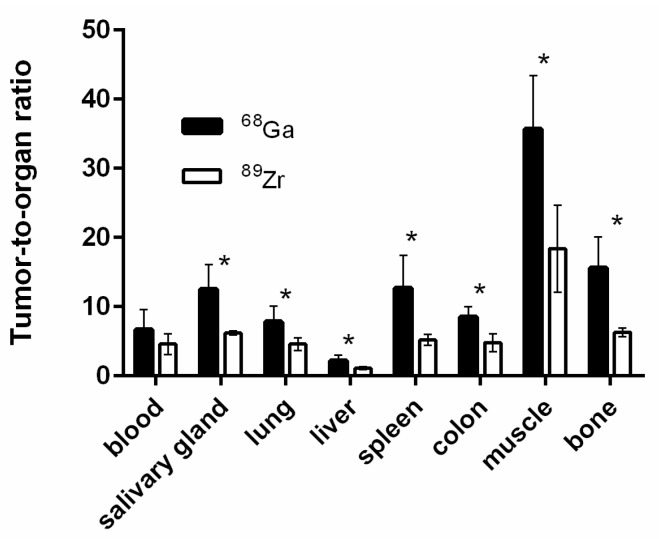
Tumor-to-organ ratios for [^68^Ga]Ga-DFO-ZEGFR:2377 and [^89^Zr]Zr-DFO-ZEGFR:2377 conjugates in BALB/C nu/nu mice bearing EGFR-expressing A431 xenografts 3 h after injection. The data were presented as the average (n = 7) and SD. Error bars might not be seen because they are smaller than the symbols. Asterisk (*) marks significant (*p* < 0.05) difference.

Figure 7 shows a comparison of the biodistribution of [^68^Ga]Ga-DFO-ZEGFR:2377 and [^89^Zr]Zr-DFO-ZEGFR:2377 conjugates 3 h after injection in BALB/C nu/nu mice bearing EGFR-expressing A431 xenografts. The blood concentration values for [^68^Ga]Ga-DFO-ZEGFR:2377 and [^89^Zr]Zr-DFO-ZEGFR:2377 were 1.4 ± 0.3 and 1.2 ± 0.2% ID/g 3 h after injection, which suggests quite a rapid blood clearance. [^68^Ga]Ga-DFO-ZEGFR:2377 demonstrated high radioactivity accumulation in the kidneys (268 ± 31 % ID/g), similarly to [^89^Zr]Zr-DFO-ZEGFR:2377 (273 ± 63 % ID/g). Tumor uptake values for [^68^Ga]Ga-DFO-ZEGFR:2377 and [^89^Zr]Zr-DFO-ZEGFR:2377 were 8.6 ± 2.4 and 5.1 ± 0.6 % ID/g, respectively. The tumor uptake of [^68^Ga]Ga-DFO-ZEGFR:2377 was significantly (*p* < 0.01) higher than the uptake of its ^89^Zr-labeled counterpart. The tumor uptake of [^68^Ga]Ga-DFO-ZEGFR:2377 exceeded the uptake in other organs except kidneys. Furthermore, [^68^Ga]Ga-DFO-ZEGFR:2377 showed significantly (*p* < 0.05) lower uptake in bone and spleen. There was a tendency to lower uptake of [^68^Ga]Ga-DFO-ZEGFR:2377 in liver, but the difference with the hepatic uptake of [^89^Zr]Zr-DFO-ZEGFR:2377 was not significant. Significant (*p* < 0.05) increase of uptake in tumor for [^68^Ga]Ga-DFO-ZEGFR:2377 caused significantly (*p* < 0.05) higher tumor-to-organ ratios (Figure 8). For example, tumor-to liver ratio for [^68^Ga]Ga-DFO-ZEGFR:2377 was 2.2 ± 0.8, which was significantly (*p* < 0.05) higher than the value for [^89^Zr]Zr-DFO-ZEGFR:2377 (1.1 ± 0.2). This suggested that better contrast of imaging for [^68^Ga]Ga-DFO-ZEGFR:2377 should provide better sensitivity of clinical imaging 3 h after injection.

**Figure 9:**
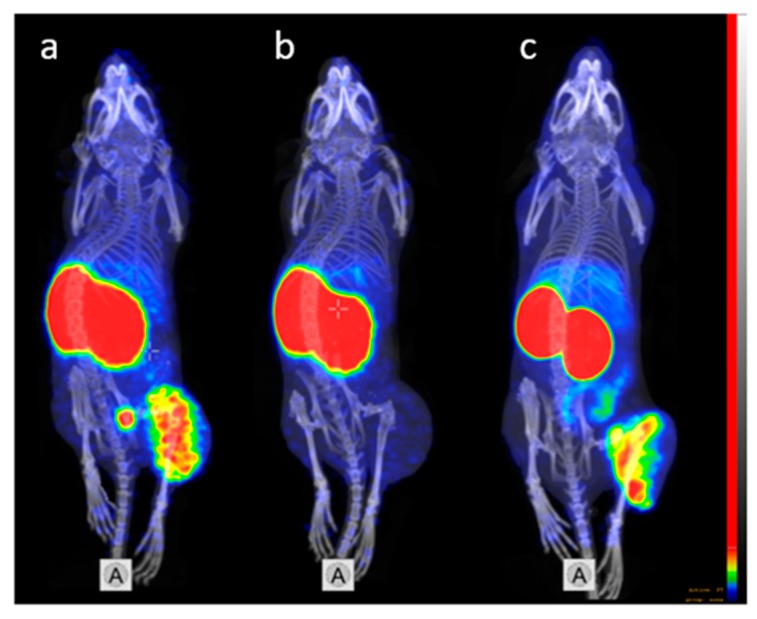
Micro PET/CT imaging of EGFR-expression in A431 xenografts using (**a**) [^68^Ga]Ga-DFO-ZEGFR:2377 and (**c**) [^89^Zr]Zr-DFO-ZEGFR:2377 at 3 h post injection. The mice were injected with 5 MBq (38 μg) of conjugates. To confirm the EGFR-specificity of [^68^Ga]Ga-DFO-ZEGFR:2377 accumulation in xenografts, receptors were saturated in one animal (**b**) by subcutaneously injection of 10 mg of cetuximab at 24 h before injection of [^68^Ga]Ga-DFO-ZEGFR:2377. The color scale has been adjusted to provide clear visualization of tumors.

The capacity of [^68^Ga]Ga-DFO-ZEGFR:2377 to specifically visualize EGFR-expressing xenografts was confirmed using microPET/CT (Figure 9) Two mice bearing A431 xenografts were imaged 3 h after injection of [^68^Ga]Ga-DFO-ZEGFR:2377. EGFR-expressing xenografts on the right hind leg were clearly visualized after 3 h using [^68^Ga]Ga-DFO-ZEGFR:2377 (Figure 9a). The tumor-to-liver and tumor-to-intestines ratios for [^68^Ga]Ga-DFO-ZEGFR:2377 were obviously higher than the ratios for [^89^Zr]Zr-DFO-ZEGFR:2377 (Figure 9c).

In the control mouse, EGF receptors were saturated by preinjection of a large molar excess of the antibody cetuximab, resulting in reduced activity uptake (Figure 9b). The imaging results were in very good agreement with the biodistribution data. The only normal organ with a high radioactivity accumulation was the kidneys. Otherwise, the uptake of radioactivity in the unblocked tumor appreciably exceeded uptake in any normal organ, providing a high-contrast image of EGFR-expressing xenograft.

## 4. Discussion

Clinical potential of visualization of EGFR expression in tumors depends on sensitivity of such diagnostics. An increase in tumor-to-organ ratios, which are provided by an imaging probe, increases the contrast of imaging and results in its improved sensitivity. In particular, good tumor-to-organ ratios are essential for major metastatic sites, such as lung, liver, and bones. Small size and high affinity of affibody molecules are important preconditions for high-contrast imaging [49]. However, the binding properties of a protein scaffold are not the only factors determining the uptake of a tracer in both tumors and normal tissues. Coupling of a radionuclide is typically associated with an attachment of a chelator or prosthetic group to a targeting protein. Such coupling modifies the properties of the surface of the probe (distribution of charge and “lipophilic patches”), which may have a strong influence on both on-target and off-target interactions of the probe in vivo and, accordingly, on the imaging contrast [1]. This prompted us to search for an optimal combination of a chelator and radionuclide for labeling of affibody-based imaging probes. 

Our previous studies [35,36] suggested that the combination of the positron-emitting radionuclide ^89^Zr and the DFO chelator for high-temperature labeling of anti-EGFR ZEGFR:2377 affibody molecule provides a PET tracer with favorable imaging properties. The use of the long-lived ^89^Zr permitted imaging at 24 h pi, which increased tumor-to-blood ratio. However, there was no improvement in tumor-to-liver and tumor-to-lung ratio, and the tumor-to-bone ratio was lower at this time-point compared to 3 h pi. For this reason, the use of ^68^Ga-DFO label seemed to be a reasonable alternative to ^89^Zr-DFO.

This study demonstrated that DFO-ZEGFR:2377 can be efficiently labeled with ^68^Ga at both room temperature and 85 °C, providing an apparent specific activity of 2 MBq/μg and radiochemical purity of 98%, which is compatible with clinical applications (Table 1). The label was stable under challenge with 1000-fold molar excess of EDTA (Table 2). The binding of [^68^Ga]Ga-DFO-ZEGFR:2377 to EGFR-expressing cell lines in vitro was proportional to EGFR expression. The binding of [^68^Ga]Ga-DFO-ZEGFR:2377 to the cells was significantly reduced by saturation of the receptors with an excess of nonlabeled ZEGFR:2377 or anti-EGFR antibody cetuximab but not by non-EGFR-specific affibody molecule or antibody, which demonstrated that the EGFR specificity of binding was preserved after labeling (Figure 4). Internalization rate of peptides labeled with different nuclides using different chelators might differ considerably [50,51,52]. This was not the case for [^68^Ga]Ga-DFO-ZEGFR:2377. The internalization rate of [^68^Ga]Ga-DFO-labeled variant was low (Figure 5) similarly to [^89^Zr]Zr-DFO-ZEGFR:2377 [36], [^68^Ga]Ga-DOTA-ZEGFR:2377 and [^57^Co]Co-DOTA-ZEGFR:2377 [32].

Saturation of EGFR in A431 xenografts resulted in highly significant (*p* < 0.001) reduction in the [^68^Ga]Ga-DFO-ZEGFR:2377 uptake in tumors (Figure 6 and Figure 9). This confirmed that EGFR-specific targeting was preserved in vivo. It has to be noted that cetuximab binds to human EGFR but not to murine EGFR, and there was no saturation of the uptake in other EGFR-expressing tissues. Furthermore, substitution of ^89^Zr by ^68^Ga resulted in significant increase in radionuclide accumulation in tumors and significant decrease in accumulation in spleen and bones. This resulted in significantly higher tumor-to-lung, tumor-to-liver, and tumor-to-bone ratios for [^68^Ga]Ga-DFO-ZEGFR:2377 (Figure 8), which creates preconditions to improved sensitivity in imaging of frequently encountered pulmonary, hepatic, and bone metastases. Besides, higher tumor-to-colon and tumor-to-lung, as well as tumor-to-salivary gland and tumor-to-muscle ratios may improve imaging of primary colorectal, lung, and head-and-neck cancers, where elevated expression of EGFR has prognostic and predictive value. The exact mechanism providing higher tumor uptake of [^68^Ga]Ga-DFO-ZEGFR:2377 compared to uptake of [^89^Zr]Zr-DFO-ZEGFR:2377 is not completely clear. The major difference is that the [^68^Ga]Ga^3+^-DFO complex has an extra negative charge compared to the [^89^Zr]Zr^4+^-DFO complex. It is known that charge is one of the factors influencing extravasation of proteins [53]. It is possible that [^89^Zr]Zr-DFO-ZEGFR:2377, carrying an additional positive charge at C-terminus, extravasates into tumors less efficiently than [^68^Ga]Ga-DFO-ZEGFR:2377. Such hypothesis is in agreement with observations for the anti-HER2 affibody molecule ZHER2:2395. ZHER2:2395 had appreciably higher uptake in SKOV-xenografts when labeled with ^68^Ga using monoamide derivatives of DOTA and NODAGA (1,4,7-triazacyclononane,1-glutaric acid-4,7-acetic acid), providing a neutral complex (15 ± 2 and 15 ± 7 %ID/g at 1 h pi, respectively) [54], compared to when it was labeled using monoamide derivative of NOTA (1,4,7-triazacyclononane-N,N′,N″-triacetic acid), providing a complex with the positive charge (5.6 ± 7 %ID/g at 1 h pi) [55]. 

The only organ with higher uptake than tumor was the kidney. High reabsorption in proximal tubuli of kidneys is typical for affibody molecules [56] as well as for protein imaging probes based on other scaffolds, such as DARPins (designed ankyrin repeat proteins) [57], ADAPTs (albumin binding domain derived affinity proteins) [58], and adnectins [59]. However, the clinical experience with anti-HER2 affibody molecules shows that this high renal reabsorption does not prevent efficient molecular imaging of targets in lumber area, including adrenal metastases [60,61]. Importantly, the use of the short-lived ^68^Ga as a label enables appreciable reduction of absorbed dose to kidneys. 

The most essential achievement of the use of [^68^Ga]Ga-DFO-ZEGFR:2377 is the positive contrast to liver, i.e., tumor-to-liver ratio exceeding one (2.2 ± 0.8). For example, all imaging probes based on EGF have much higher uptake in liver than in tumors [21,23] or the tumor-to-liver ratio is close to one [22], which precludes the use of such tracers for imaging of EGFR expression in hepatic metastases. The same is true for affibody-based PET imaging probes. The tumor-to-liver ratios are usually either lower than one for [^68^Ga]Ga-DOTA-ZEGFR:2377 [32], [^18^F]AlF-NOTA-ZEGFR:2377 [31] or are very close to one, as for [^18^F]F-CBT-ZEGFR:1907 [31] or [^18^F]F-FBEM-ZEGFR:1907 [30]. In addition, [^68^Ga]Ga-DFO-ZEGFR:2377 provided appreciably higher tumor-to-bone ratio (16 ± 5) compared to [^18^F]F-CBT-ZEGFR:1907 (0.32 ± 0.07) or [^18^F]AlF-NOTA-ZEGFR:2377 (2.81 ± 0.62) [31]. The only affibody-based tracer that provides similar or slightly better tumor-to-liver (3.1 ± 0.5) and tumor-to-bone (24 ± 5) ratios is [^55^Co]Co-DOTA-ZEGFR:2377 [32]. It should be taken into account that production of ^55^Co is feasible using low energy cyclotron installed at PET centers. However, production of ^55^Co is currently established at only two research centers: University of Wisconsin–Madison and University of Southern Denmark, Odense. By contrast, ^68^Ga is widely accessible due to its production by the commercially available ^68^Ga/^68^Ga generator. We consider that clinical translation of [^68^Ga]Ga-DFO-ZEGFR:2377 would therefore be more facile compared with the translation of [^55^Co]Co-DOTA-ZEGFR:2377.

The results of this study, as well as results of previous studies on affibody molecules, indicate an essential issue in development of scaffold protein-based imaging probes. The labeling strategy must provide a rapid and efficient coupling of a radionuclide to a targeting vector and high stability of the label in vivo. However, both chelator–radionuclide complex and a linker for its conjugation to a targeting protein may have a substantial influence on biodistribution and targeting properties of a conjugate. The difference in positioning of chelator or the conjugation chemistry may be decisive. Moreover, advantages of rapid and stable chelation might not always compensate disadvantages of poor distribution profile. For example, macrocyclic triaza chelator NOTA (2-[4,7-bis(carboxymethyl)-1,4,7-triazonan-1-yl]acetic acid) is one of the best chelators for Ga, providing a stability constant (K_ML_) of 31.1 [62]. In the case of placement at N-terminus of synthetic anti-HER2 ZHER2:S1 affibody molecules, the use of monoamide of NOTA derivative NODAGA results in the ^68^Ga-labeled conjugate providing higher tumor-to-blood ratios than conjugates labeled using monoamides of DOTA and NOTA [63]. [^68^Ga]Ga-NOTA-ZHER2:S1 has the same tumor-to-blood ratio as [^68^Ga]Ga-DOTA-ZHER2:S1 but significantly lower tumor-to-liver and tumor-to-muscle ratios [63]. As has been mentioned above, recombinantly produced ZHER.2395 labeled with ^68^Ga at C-terminus via maleimido derivatives of NODAGA and DOTA outperformed the variant labeled using NOTA [54,55]. Overall, the use of NODAGA providing an additional negative charge seems to be more promising than the use of NOTA when the same conjugation chemistry and position of the label is used. Another promising derivate of NOTA is the backbone-modified, benzylisothiocyanate-containing *p*-SCN-Bn-NOTA (2-*S*-(4-Isothiocyanatobenzyl)-1,4,7-triazacyclononane-1,4,7-triacetic acid). The use of this chelator for labeling of PSMA-binding dipeptides provided excellent tumor-to-blood ratios [64]. However, this chelator is not suitable for site-specific labeling of affibody molecules. It is conjugated to amino groups. However, the affibody scaffold contains seven amino groups (six lysines and N-terminal amino group). Therefore, coupling of *p*-SCN-Bn-NOTA to an affibody molecule would result in a mixture of conjugates with different label positions and different degree of modification. Thus, comparative studies are necessary to select the best imaging probe.

In conclusion, [^68^Ga]Ga-DFO-ZEGFR:2377 is stable, binds specifically to EGFR in vitro and in vivo, and provides the best reported contrast in preclinical imaging of EGFR-expressing tumors, thus making it a promising candidate for clinical translation. Another important lesson from this study is that careful optimization of molecular design, including the combination of radionuclide and chelator, enables a probe providing the best contrast for molecular imaging of therapeutic targets to be obtained. 

## Figures and Tables

**Table 1:** Labeling of DFO-ZEGFR:2377 using gallium-68.

Temperature, °C	Radiochemical Yield ^1^, %	Isolated Yield ^2^, %	Radiochemical Purity, %	Maximum Apparent Specific Activity ^3^, MBq/μg
25	95 ± 4	81 ± 6	99 ± 0	2
85	77 ± 5	73 ± 3	98 ± 1	2

^1^ Radiochemical yield is determined as percentage of affibody-bound activity before purification as measured by instant thin-layer chromatography (ITLC). ^2^ Isolated yield is determined as percentage of activity in the high molecular weight fraction after NAP-5 purification; ^3^ Maximum apparent specific activity obtained at the end of purification.

**Table 2:** Stability of ^68^Ga-DFO-ZEGFR:2377 in phosphate-buffered saline (PBS) and under EDTA challenge ^1^.

Time, h	25 °C		85 °C	
	PBS	EDTA	PBS	EDTA
1	98.6 ± 0.1	98 ± 0.4	97.9 ± 0.1	97.8 ± 0.3
3	98.2 ± 0.2	97.4 ± 0.5	97.0 ± 0.7	97.1 ± 0.8

^1^ Data were calculated from ITLC measurement and are presented as average ± maximum error (n = 3).
